# Cannabis users: Screen systematically, treat individually. A descriptive study of participants in a randomized trial in primary care

**DOI:** 10.1371/journal.pone.0224867

**Published:** 2019-12-02

**Authors:** Catherine Laporte, Céline Lambert, Bruno Pereira, Olivier Blanc, Nicolas Authier, David Balayssac, Georges Brousse, Philippe Vorilhon

**Affiliations:** 1 Département de Médecine Générale, Equipe d’Accueil 7280, Unité de Formation et de Recherche de Médicine, Université Clermont Auvergne, Clermont-Ferrand, France; 2 Délégation à la recherche clinique et à l’innovation, Centre Hospitalier Universitaire Clermont-Ferrand,Clermont-Ferrand, France; 3 Equipe d’Accueil 7280, Unité de Formation et de Recherche de Médicine, Université Clermont Auvergne; Service de Psychiatrie B et d’addictologie, Centre Hospitalier Universitaire de Clermont-Ferrand, Clermont-Ferrand, France; 4 Inserm, Neuro-Dol, Université Clermont Auvergne; Service de Pharmacologie Médicale, Centres Addictovigilance / Pharmacovigilance, Centre Evaluation et Traitement de la Douleur, Centre Hospitalier Universitaire de Clermont-Ferrand, Clermont-Ferrand, France; 5 Inserm U1107, NEURO-DOL, Unité de Formation et de Recherche de Pharmacie, Université Clermont Auvergne; Laboratoire de Toxicologie, Délégation à la recherche clinique et à l’innovation, Centre Hospitalier Universitaire de Clermont-Ferrand, Clermont-Ferrand, France; 6 Université Clermont Auvergne, CNRS, SIGMA Clermont, Institut Pascal, France; University of the Witwatersrand, SOUTH AFRICA

## Abstract

**Purpose:**

The aim of the present study was to describe the profiles of a sample of young cannabis users not seeking care, for use in general practice in France.

**Methods:**

In this cross-sectional study, baseline data were used from a previous clinical randomized trial, in which a brief intervention was tested. The participants were 262 cannabis users aged 15 to 25 years who smoked at least one joint per month. Assessment was undertaken both by the GP and via an anonymous self-reporting questionnaire. All statistical analyses were performed using Stata software and R. We used multiple correspondence analysis to determine the profiles of users.

**Results:**

Among the 262 patients, 46.2% were daily users (more than 30 joints per month), 25.6% were regular users (from 10 to 29 joints per month), and 28.2% were recent users (fewer than 10 joints per month). The higher the frequency of use, the greater the incidence of unaccompanied use, daily use and week use *(p from <0*.*001 to 0*.*01)*. The motivations of daily users were mostly self-treatment and habit (*p <0*.*05*). The cannabis abuse screening trial score revealed risky use for 87.5% of daily users and 34.4% for recent users. Factorial analysis identified 5 profiles according to age, risk, and motive for use. The reasons for consultation were equally distributed among users regardless of their level of use or their profile (*p > 0*.*05*).

**Conclusions:**

The results provide support for the practice of asking young patients systematically about their cannabis use, allowing GPs to identify users who require medical care. GPs should consider the differences between participants according to their profile in order to determine the appropriate type of care.

**Trial registration:**

Clinicaltrials.gov NCT01433692.

## Introduction

Cannabis is a commonly used substance worldwide, with 192 million people reporting having used it at least once per year [1]. France has among the highest levels of use in Europe [2]; in 2014, 25% of French adolescents (15–18-year-olds) and 17% of young adults (18–25-year-olds) were monthly users [3][4]. Early onset of cannabis use increases the risk of subsequent development of substance-use disorders [5], and is associated with poor mental health including depression and suicidality [6], psychotic disorders [7], and neurocognitive decline with low performance at school [8]. Such problems have been found even in adolescents who had used cannabis only once or twice in their lives [9]. Identification of factors related to early screening of cannabis is key to the prevention of these adverse effects.

In France, 80% of 15–25-year-olds consulted their general practitioners (GPs) at least once in the previous year [10]. In Switzerland (a country with universal insurance coverage like France), it was shown that young people who engaged in health-compromising behaviors accessed GPs to the same extent as others who did not [11]. While it is clear that GPs could be privileged interlocutors for adolescents to talk about cannabis use, only 8% of French GPs ask their patients about their cannabis use and just 2% use specific questionnaires to identify disorders related to cannabis [12], even though there are many available for adolescents (CAST) [13]. There is thus a clear need for better methods of facilitating monitoring by GPs of young cannabis users.

In 2014, the authors conducted a randomized control trial to test a brief intervention (BI) to be performed by GPs on young cannabis users (15–25 years) in primary care in France. This study was referred to as CANnabis And young users: a Brief Intervention to reduce their Consumption (CANABIC) [14]. The intervention was an interview designed according to the feedback, responsibility, advice, menu, empathy, self-efficacy (FRAMES) model [15].

The aim of the current study is therefore to use multiple correspondence analysis to describe the profiles of a sample of young cannabis users not seeking care for use in general practice in France. We hypothesize that by analyzing the characteristics of the 262 participants, it may be possible to identify some characteristics that could help GPs to screen users and to adapt their advice on prevention to the patient at hand.

## Method

For this cross-sectional study, baseline data were obtained from a previous clinical cluster-randomized controlled trial of a brief intervention [14]. We used the STROBE cross-sectional checklist when writing our report (S1 Table) [16].

### Participants

The participants were the 262 adolescents and young adults involved in the CANABIC study from April 2014 to April 2015. All GPs working in three areas of France (Auvergne, Rhône-Alpes, and Languedoc-Roussillon) were invited by mail to take part in the trial. Their addresses were sourced from the National Institute for Statistics and Economic Studies.

### Inclusion criteria

Participants had to be 15 to 25 years old and to have used at least one joint per month for at least one year. Participants were not included in the study if they were suffering from a psychotic disorder or were receiving ongoing treatment for an addiction.

### Design

The GP was the unit of randomization [17]. The randomization was carried out using Stata version 12 (StataCorp, College Station, USA). GPs registered in the intervention group conducted an interview using the BI model, which is defined in six key stages using FRAMES [15] during each consultation at inclusion, and after 3 and 6 months. Investigators registered in the control group provided care according to their usual practice.

### Screening and assessment

In both groups, GPs had to consult all patients from 15 to 25 years on their own, and to ask them whether they used cannabis and how much, regardless of the reason for consultation. Then, they had to include the first five eligible candidates from those who had used at least one joint per month for at least one year. GPs were asked to participate in the study on a voluntary basis and were assured of the confidentiality of the data collected.

### Measures

As part of the baseline consultation, GPs asked participants about their sociodemographic characteristics (e.g. employment; according to the questions from Insee -National Institute for Statistics and Economic Studies-[18]), and their medical records (surgical, medical, family and psychotropic treatment—to check for psychiatric comorbidities-) (S1 Appendix). GPs have informed the reason for consultation of their patients (the answer was free). We grouped the main reasons: somatic (e.g. pain), prevention (e.g. aptitude visit for sport, vaccination), Ear Nose Throat-bronchopulmonary (e.g. cold, bronchitis), pychiatric (e.g. anxiety). GPs informed about their uses: quantity of tobacco, alcohol and cannabis use. For cannabis, they also asked if they used water pipe, hashish or weed. Following the OFDT definitions (French Observatory of Drugs and Addictions), we classified participants who smoked 30 joints per month as “Daily users”; participants who used at least 10 times in the preceding 30 days as “Regular users”; and participants who smoked fewer than 10 joints during the month preceding the survey as “Recent users” (they were “recent” but not “regular”) [4].

Then, all patients completed an anonymous self-administered questionnaire in which they provided information about their use (when and where they used, their motivations for use, and their sources of cannabis -purchase or culture-) (S2 Appendix). They also informed their perception of the consequences of their use (on their health, their personal and professional life) and if they drove after consuming. We chose to collect data describing users and to be discriminating on the severity of use. Some items were derived from a preliminary qualitative study of adolescents [19]. The personal purchase of cannabis increases while the use of weed decreases with the increase of the level of use [4]. Some substances are widely used among college students to aid sleep [20]. Finally, they completed the cannabis abuse screening trial (CAST) [21]. We used the CAST score in the self-questionnaire, to estimate the scale of cannabis-related problems. The CAST score was not assessed by the GP. We used the binary CAST validated for the health-seeking population in France [21]. There were 6 questions (score 1 for “yes”, 0 for “no”). Respondents could be classified into 3 groups: Low risk (score less than or equal to 1), Moderate risk (score equal to 2: harmful use of alcohol and other drugs, including cannabis), High risk (score greater than or equal to 3: high severity of consumption). We used the location of the GP as a proxy for the participant’s location.

### Statistical analysis

Because this was an ancillary study of a randomized trial, the sample size was determined by the study population, which comprised 262 participants. However, according to general guidelines reported by several authors for conducting factor analyses [22][23], the sample size was consistent and statistically powered to determine the profiles of the young cannabis users. All statistical analyses were performed using Stata software (version 13, StataCorp, College Station, US) and R 3.3.3 (http://cran.r-project.org/). All tests were two-sided with type I error set at 0.05. Baseline characteristics (GPs and patients) were presented as frequencies and associated percentages for categorical parameters and as the mean ± standard deviation or as median [interquartile range] for continuous data, according to statistical distribution.

Agreement between the number of joints consumed per month reported anonymously in the self-questionnaire and as presented to the GP was assessed using Lin’s concordance correlation coefficient [24], expressed with a 95% confidence interval.

The categorical variables were compared between independent groups (<10, 10 to 29, or ≥30 joints per month according to the French Observer for Drugs and Drug Addictions [4]) using the chi-squared test or the Fisher’s exact test, and followed by the Marascuilo procedure if appropriate (omnibus p-value less than 0.05). Quantitative data were compared between groups using ANOVA or Kruskal-Wallis test, as appropriate. Gaussian distribution was verified by the Shapiro-Wilk test and homoscedasticity by the Bartlett’s test. When appropriate (omnibus p-value less than 0.05), post-hoc tests were carried out: Tukey-Kramer post ANOVA and Dunn’s test post Kruskal-Wallis. Effect size (ES) were calculated and were interpreted according to Cohen’s recommendations, who defined ES as small (ES:0.2), medium (ES:0.5) and large (ES:0.8, “grossly perceptible and therefore large”) [25]. Then, multiple correspondence analysis (MCA), which can be seen as a generalization of principal component analysis for categorical rather than quantitative variables, was applied to study the associations between characteristics of patients (age, gender, employment), cannabis use (quantity of use, consumption mode, motive for consumption, etc.), and characteristics of GPs (urban or rural, to assess the origin of the participant). This exploratory method was used to determine whether there were any profiles of young people based on the levels of use, and to summarize the relationships between the variables and to detect the underlying structure of the data. For this analysis, variables were chosen according to univariate results, clinical relevance, and statistical distribution (parameters always present or always absent were not considered). Finally, only individuals without missing data were used for MCA. Then, hierarchical clustering was used to determine groups of patients according to Ward’s method. A sensitivity analysis was conducted to study the impact of missing data on results comparing the samples with and without missing data for the characteristics of the main patient.

### Ethical considerations

The protocol received a favorable assessment from the Comité de Protection des Personnes SUD-EST VI (South-East VI Committee for the Protection of Persons) of Clermont-Ferrand, on March 5, 2010. The GPs provided patients with written information about the study, and explained clearly the design and purpose of the study. Inclusion in the study was voluntary, anonymous, within medical confidentiality, and ensured an unconditional right to withdrawal. Then, according with French law, after the patients had given their verbal consent to the GPs, they signed a form of non-opposition. This form was accepted by the ethics committee, allowing minors to participate without parental consent.

## Results

### Univariate analysis

#### Sociodemographic characteristics and cannabis use

The sociodemographic characteristics of the global population according to their quantity of use are shown in Table 1. There were 169 males (64.5%), and the average age of the population was 20.6±2.6 years. The majority either worked (44.3%, n = 116) or were students (41.2%, n = 108). At baseline, participants reported smoking a median of 20 [6–60] joints per month to their GP and 20 [5–50] joints per month on the self-administered questionnaire. The concordance correlation coefficient (Lin) was 0.90, 95%CI [0.87; 0.93].

**Table 1:** Sociodemographic characteristics of the population.

		All sample (n = 262)	Group 1 Recent users <10 joints/month (n = 74)	Group 2 Regular users 10–29 joints/month (n = 67)	Group 3 Daily users ≥30 joints/month (n = 121)	*p*
Male		169 (64.5)	44 (59.5)	45 (67.2)	80 (66.1)	*0*.*56*
Age		20.6 ± 2.6	20.3 ± 2.2	19.8 ± 2.9	21.2 ± 2.3	*<0*.*001*^*b*.*c*^
Marital status: single		209/260 (81.0)	62/73 (84.9)	56/67 (83.6)	91/120 (77.1)	*0*.*34*
Living alone		63 (24.1)	17 (23.0)	17 (25.4)	29 (24.0)	*0*.*95*
**Employment**						
In work		116 (44.3)	28 (37.8)	25 (37.3)	63 (52.1)	*0*.*06*
No occupation		38 (14.5)	5 (6.8)	7 (10.5)	26 (21.5)	*0*.*01*^*b*^
Student		108 (41.2)	41 (55.4)	35 (52.2)	32 (26.5)	*<0*.*001*^*b*.*c*^
**Work**						
Employee		73/148 (49.3)	18/33 (54.6)	13/30 (43.3)	42/85 (49.4)	*0*.*67* *0*.*67* *0*.*86*
Worker		45/148 (30.4)	8/33 (24.2)	10/30 (33.3)	27/85 (31.8)	
Other		30/148 (20.3)	7/33 (21.2)	7/30 (23.3)	16/85 (18.8)	
**Medical record**						
Medical		78 (29.8)	19 (25.7)	18 (26.9)	41 (33.9)	*0*.*41*
Surgical		63 (24.1)	17 (23.0)	17 (25.4)	63 (24.1)	*0*.*23*
Family		93 (35.5)	29 (39.2)	28 (41.8)	36 (29.8)	*0*.*25*
Psychotropic treatment		10 (3.8)	3 (4.1)	3 (4.5)	4 (3.3)	*0*.*92*
**Reason for consultation**						
Somatic		92/258 (36.2)	32/71 (45.1)	20/66 (30.3)	40/117 (34.2)	*0*.*16*
Prevention		47/258 (18.5)	12/71 (16.9)	16/66 (24.2)	19/117 (16.2)	*0*.*38*
ENT-Bronchopulmonary		39/258 (15.4)	8/71 (11.3)	14/66 (21.2)	17/117 (14.5)	*0*.*26*
Psychiatric		30/258 (11.8)	9/71 (12.7)	4/66 (6.1)	17/117 (14.5)	*0*.*23*
Others		46/258 (18.1)	10/71 (14.1)	12/66 (18.2)	24/117 (20.5)	*0*.*54*
**GP work area**						
Rural		26/ 216 (12.0)	7/63 (11.1)	5/56 (8.9)	14/97 (14.4)	*0*.*58*
Semi-rural		85/216 (39.4)	28/63 (44.4)	24/56 (42.9)	33/97 (34.0)	*0*.*35*
Urban		105/216 (48.6)	28/63 (44.4)	27/56 (48.21)	50/97 (51.6)	*0*.*68*

Data are presented as frequencies (associated percentages) or as mean ± standard deviation. ENT = Ear Nose & Throat; GP = general practitioner; a = p<0.05 between Group 1 and Group 2, b = p<0.05 between Group 1 and Group 3, c = p<0.05 between Group 2 and Group 3.

Nearly half of the participants (46.2%, n = 121) were daily users (more than 30 joints per month), the remainder being divided into regular users (from 10 to 29 joints per month; 25.6%, n = 67) and recent users (fewer than 10 joints per month; 28.2%, n = 67). Daily users were one year older than other users (21.2±2.3 vs 19.8±2.9, *p<0*.*001* for regular users and 20.3±2.2, *p = 0*.*05*, for recent users), and there was no difference between regular and recent users (*p = 0*.*66*). There were no other differences in sociodemographic characteristics by cannabis use except for employment status. Recent or regular users were more often students (55.4%, n = 41 and 52.2%, n = 35 respectively) than daily users (26.5%, n = 32, *p<0*.*001* for both), and there was no difference between recent and regular users (*p = 0*.*71*). Health history and reasons for consultation were independent of the quantity of use (*p>0*.*05*). There was no difference in the number of joints used per month between males, 20 [8–60], and females, 20 [5–60] (*p = 0*.*56*).

#### History and patterns of cannabis use

As shown in Table 2 (or additional files), the average age of onset of use was 15.1±1.9 years. Recent users started to use one year later (15.7±1.6) than daily and regular users (14.9±1.6, *p = 0*.*001*and 14.9±2.0, *p = 0*.*03*), and there was no difference between regular and daily users (*p>0*.*05*). Almost all users smoked tobacco (91.6%, n = 240) and 77.9% (n = 204) had consumed alcohol in the previous month, but there were no differences among levels of cannabis use. Daily users had experimented with cocaine more often than others (40.5% (n = 49) versus 17.9% (n = 13) for recent and 16.11% (n = 14) for regular users, *p<0*.*001* for both).

**Table 2:** Description of cannabis use among daily, regular and recent user groups.

		All sample (n = 262)	Group 1 Recent users <10 joints/month (n = 74)	Group 2 Regular users 10–29 joints/month (n = 67)	Group 3 Daily users ≥30 joints/month (n = 121)	*p*
**GP questionnaire**						
Age at onset of cannabis use		15.2 ± 1.9	15.7 ± 1.6	14.9 ± 1.6	14.9 ± 2.0	*0*.*005*^*a*.*b*^
Water pipe		39/260 (15.0)	6/74 (8.1)	15/67 (22.4)	18/119 (15.1)	*0*.*06*
Weed		213 (81.3)	55 (74.3)	55 (82.1)	103 (85.1)	*0*.*17*
Hashish		174 (66.4)	44 (59.5)	44 (65.7)	86 (71.1)	*0*.*25*
**Self-questionnaire**						
**Consumption mode**						
Alone		119/216 (55.1)	9/63 (14.3)	28/56 (50.0)	82/97 (84.5)	*0*.*001*^*a*.*b*.*c*^
Week		148/216 (68.5)	19/63 (30.2)	37/56 (66.1)	92/97 (94.8)	*0*.*001*^*a*.*b*.*c*^
During the day		75/216 (34.7)	13/63 (20.6)	14/56 (25.0)	48/97 (49.5)	*0*.*001*^*b*.*c*^
**Place of consumption**						
Home		169/216 (78.2)	36/63 (57.1)	34/56 (78.6)	89/97 (91.8)	*<0*.*001*^*a*,*b*^
Friends house		187/216 (86.6)	56/63 (88.9)	51/56 (91.1)	80/97 (82.5)	*0*.*26*
Workplace		18/216 (8.3)	1/63 (1.6)	7/56 (12.5)	10/97 (10.3)	*0*.*06*^*b*^
Clubbing		51/216 (23.6)	5/63 (7.9)	19/56 (33.9)	27/97 (27.8)	*0*.*002*^*a*.*b*^
**Motive for consumption**						
Relaxing		179/216 (82.9)	45/63 (71.4)	47/56 (83.9)	87/97 (89.7)	*0*.*01*^*b*^
Partying		112/216 (51.9)	43/63 (68.3)	31/56 (55.4)	38/97 (39.2)	*0*.*001*^*b*^
Sleep		69/216 (31.9)	9/63 (14.3)	14/56 (25.0)	46/97 (47.4)	*<0*.*001*^*b*.*c*^
Habit		63/216 (29.2)	3/63 (4.8)	13/56 (23.2)	47/97 (48.5)	*<0*.*001*^*a*.*b*.*c*^
Reduce an anxiety		59/216 (27.3)	12/63 (19.0)	13/56 (23.2)	34/97 (35.1)	*0*.*06*
**Origin of cannabis**						
Culture		28/196 (14.3)	4/59 (6.8)	6/47 (12.8)	18/90 (20.0)	*0*.*07*^*b*^
Buying		164/210 (78.1)	36/62 (58.1)	48/55 (87.3)	80/93 (86.0)	*<0*.*001*^*a*.*b*^
**Others drugs**						
Psychotropic medications		10 (3.8)	3 (4.1)	3 (4.5)	4 (3.3)	*0*.*92*
Alcohol use past month		204 (77.9)	63 (85.1)	51 (76.1)	90 (74.4)	*0*.*20*
Tobacco use past month		240 (91.6)	70 (94.6)	64 (95.5)	106 (87.6)	*0*.*10*
Cocaine experimentation		73/261 (28.0)	13/73 (17.8)	11/67 (16.4)	49/121 (40.5)	*<0*.*001*^*c*^
Heroin experimentation		15/261 (5.8)	3/73 (4.1)	5/67 (7.5)	7/121 (5.8)	*0*.*70*
**Perception of consequences**						
On health		167/214 (78.0)	46/63 (73.0)	43/55 (78.2)	78/96 (81.3)	*0*.*47*
On personal life		118/214 (55.1)	34/63 (54.0)	22/56 (39.3)	62/95 (65.3)	*0*.*008*^*c*^
On professional/scholarly life		130/213 (61.0)	35/63 (55.6)	29/56 (51.8)	66/94 (70.2)	*0*.*05*
Driving after use		112/212 (52.8)	19/60 (31.7)	24/55 (43.6)	69/97 (71.1)	*<0*.*001*^*a*.*b*^
**CAST**						
No risk (≤1)		30/212 (14.1)	24/61 (39.3)	5/55 (9.1)	1/96 (1.0)	*0*.*06*
Moderate risk (2)		36/212 (17.0)	16/61 (26.2)	9/55 (16.4)	11/96 (11.5)	*<0*.*001*^*a*.*b*^
Daily risk (≥3)		146/212 (68.9)	21/61 (34.4)	41/55 (74.5)	84/96 (87.5)	*0*.*005*^*a*.*b*^

Data are presented as frequencies (associated percentages) or as mean ± standard deviation; CAST = Cannabis abuse screening trial; a = p<0.05 between Group 1 and Group 2, b = p<0.05 between Group 1 and Group 3,c = p<0.05 between Group 2 and Group 3

The higher the frequency of use, the more the use was unaccompanied, during the week, at home, and out of habit *(p from<0*.*001 to 0*.*01)*. The motivations for use were mostly festive for recent users (*p from <0*.*001 to 0*.*05*) and self-therapeutic for daily users, such as for relaxation or sleep (*p from <0*.*001 to 0*.*006)*. Recent users purchased their own cannabis less often than regular or daily users (58.1%, n = 36 vs 87.3%, n = 48 and 86%, n = 80, *p<0*.*001* for both, without difference between regular and daily users, *p = 0*.*83*). Recent users cultivated their own product less than daily users (6.8%, n = 4 vs 20%, n = 18, *p = 0*.*03*). A majority of participants (78.0%, n = 167) perceived consequences of their use on their health, but there were no differences among levels of use. Daily users had a stronger perception of the personal and professional consequences (respectively 65.3%, n = 62 and 70.2%, n = 66) of their cannabis use than regular users (39.3%, n = 22, *p = 0*.*002* and 51.8%, n = 29, *p = 0*.*02*). More than half of all participants (52.8%, n = 112) reported driving after using cannabis, but daily users were the most concerned (71.1%, n = 69) compared with recent and regular users (respectively 31.7%, n = 19 and 43.6%, n = 24, *p<0*.*001* for both).

### Multiple correspondence analysis

The CAST score revealed the risk of consumption for the majority of daily users (87.5%, n = 84) and regular users (74.6%, n = 41). There was a high correlation between the number of joints smoked per month and the CAST score (r = 0.60, *p<0*.*001*). However, 34.4% of recent users (n = 21) had a risky level of consumption as indicated by a high CAST score (>3). We decided to consider the CAST instead of the number of joints per month because it was a better indicator of the severity of consumption. For the MCA, 63 out of 262 (24%) subjects were removed because of missing data, and 199 were retained. These two samples are not significantly different in terms of any parameters selected for analysis. The variables that contributed most to the formation of the first axis (X axis) were age during the study, age of onset, and use of weed. The variables that contributed most to the formation of the second axis (Y axis) were the method and the risk of use, including lone use, use during the week, use during the day, smoke to sleep, smoke out of habit, and CAST score. Vector analysis identified which variables were inter-related and thus allowed us to define 5 clusters of patients.

**Fig 1:**
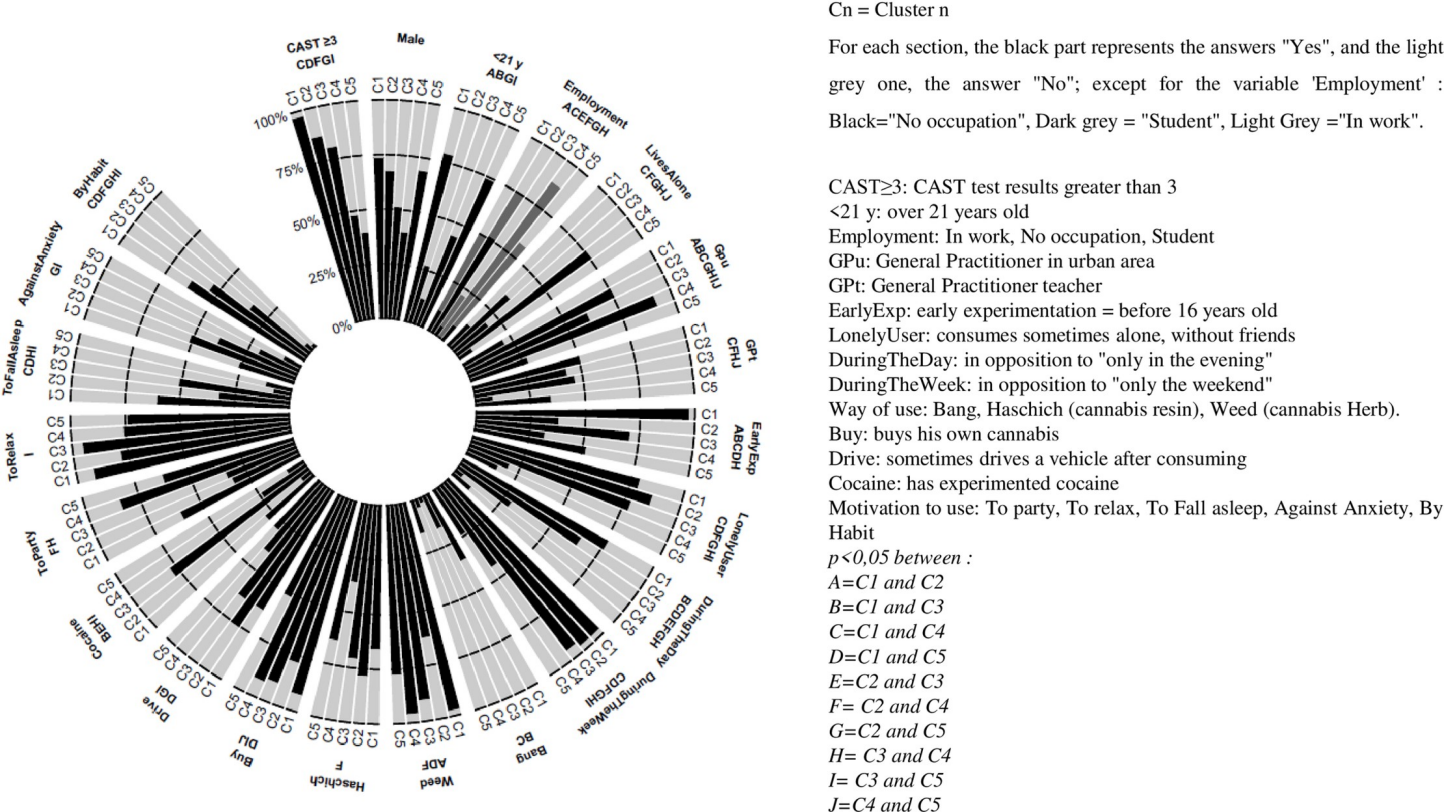
Distribution of each criterion from Multiple Correspondence Analysis (MCA) by clusters of users.

Fig 1 shows the distribution of each criterion used for the MCA in the 5 clusters. The clusters are described below according to the variables of interest, which are the significantly different variables among the clusters. Cluster 1 (C1, n = 38), termed “Risky Adolescent users”, includes young people (<21 years old) with a young age of onset of use (<16 years old), who smoked a median of 33.5 [15–90] joints per month, during the day, on weekdays, and alone, who had a CAST ≥3, who smoked weed, and who bought cannabis. In addition, they attended GPs in rural areas. Cluster 2 (C2, n = 37), termed “Risky young unemployed users”, includes subjects who smoked a median of 45 [25–100] joints per month, on weekdays and alone, who had a CAST ≥3 and who did not consume cocaine. In addition, nearly two thirds of unemployed people belong to this cluster, and three quarters of this cluster attended GPs in urban areas. Cluster 3 (C3, n = 41) is termed “Risky young worker users”, and includes subjects over 21 years old who smoked a median of 30 [20–60] joints per month, used on weekdays to relax, used alone more often (for 78% of them), and bought their own cannabis. They used cocaine and did not use a water pipe. They were professionally active. Cluster 4 (C4, n = 25), termed “Low risk student users”, includes subjects who smoked a median of 5 [3–10] joints per month, in the evenings at parties. They did not use alone, or use routinely, or to aid sleep. They were students and mostly lived alone, and attended GPs in urban areas. Cluster 5 (C5, n = 58), termed “Low risk Adolescent users”, includes subjects under 21 years old, who smoked a median of 6 [3–20] joints per month, did not consume alone, during the day or during the week. They did not consume routinely, to aid sleep or to reduce anxiety. They did not consume cocaine and did not live alone. The majority of people in this cluster attended GPs in rural areas.There was no difference in male:female ratio between clusters (*p>0*.*05* for each comparison) and no difference in the reason for consultation (*p = 0*.*31)*. Participants in C1 and C2 more commonly received psychotropic treatment than the other groups (7.9% (3/38) and 13.5% (5/37), versus none for C3 and C4 and 3.4% (2/58) for C5, *p = 0*.*04*). The main criteria are summarized in a Supporting Information (S2 Table).

## Discussion

The aim of our study was to help GPs how to screen cannabis users and to adapt their prevention advice for each patient. By analyzing the baseline data of a randomized controlled trial in primary care, we showed that when GPs systematically screened cannabis use among their patients from 15 to 25 years, we can’t identified only one profile of users, but 5 different profiles: Low risk Adolescent users, Risky Adolescent users, Low risk student users, Risky young worker users and Risky young unemployed user. This means that GPs must interview all adolescents to identify cannabis users, without the need for a priori assumptions. On the other hand, among the 5 groups, the level of risk was not the same, nor the motivations to consume, which can allow the GPs to identify more easily risky users and choose the care to propose to the patient according to his risk profile. To date, this study is the first to describe young cannabis users in primary care who are not being monitored for a substance use disorder.

By screening their patients in routine care, a representative sample of GPs [14] identified 262 cannabis users, among whom 46.2% were daily users (more than 30 joints per month), 25.6% were regular users (from 10 to 29 joints per month), and 28.2% were recent users., and 85.9% (n = 182) had a moderate-to-high risk related to their use and therefore needed either a BI or an addiction consultation [26] according to the DSM-V criteria [27]. These users smoked 20[6–60] joints per month on average, while the impact of cannabis use on cognitive functioning can be observed at 17.5 joints per month [28]. GPs believe that adolescents are not necessarily going to answer their questions truthfully [29], but Lin’s concordance correlation coefficient showed that the answers were the same at the GP’s surgery or via anonymous self-administered questionnaire. Even if they doubt their role [29], the prevalence of cannabis use among these patients and the impact on health must convince GPs of the need to question patients on their cannabis habit.

The MCA revealed that rather than there being one sole type of cannabis user, there were five different clusters. Age, professional status, and psychotropic medication varied between groups. Analysis of a sample of young Americans in an outpatient substance abuse treatment program for cannabis problems and included in a randomized trial also showed heterogeneity among cannabis users, particularly in terms of increased problems with social functioning, more mental health issues, and a requirement for individually tailored care [30]. An Australian study showed differences between boys and girls in the level of severity and method of use [31]. Following a consultation an adolescent can be placed in one of the five clusters. This classification does not make it any easier for GPs to identify users versus non-users: GPs must continue to interview all adolescents to identify cannabis users, without the need for a priori assumptions. This screening is important because primary care health professionals may have a negative view of substance users, which can affect the quality of care [32]. On the other hand, three groups showed high risk in their CAST score (38 Risky Adolescent users, 37 Risky young unemployed users and 41 Risky young worker users) and had common characteristics: they had smoked before they were 16 years old, they smoked more than 30 joints per month, alone, during the week, in the day, and take psychoactive treatments. This classification makes it easier for GPs to identify risky users when meeting a teenager who belongs to one of these 3 profiles.

Depending on the level of use or the cluster, there were differences in motivation, perception of risk, and medical history. Regular users showed better perception of the repercussions of their use in their professional life than daily users, who may have felt less concern. The GP could, therefore, use a brief intervention [15] to inform regular users of the consequences and repercussions of their substance use. To address this topic for daily users probably has less impact on their behaviors because they are already aware of the risks involved. However, motivations for use differed among clusters: Risky Adolescents users (C1) and Risky young unemployed users (C2) mostly smoked for self-treatment (relaxation and sleep), and also more often received psychotropic treatment. Given the interplay between substance use and vulnerability to mental health disorders [33], identification of symptoms of anxiety or depression in those clusters may be an issue for the care of these individuals. Finally, risk-taking was also different: the first 3 groups drove more often after using cannabis. We found that cocaine was the preferred drug of Risky young unemployed users. These elements show that the identification of risk can allow the GP to provide appropriate care for each level of use or for a profile of a given user.

The problem for GPs is one of how and when to screen young patients [29]. The average duration of study visits (inclusion and follow-up) was 20 minutes. Given the multiplicity of reasons for consultation during the inclusion process, it can be concluded that all possible moments are good for interviewing teenagers about their cannabis use and that this does not take much time. One of the distinguishing features of the profiles is the CAST, which is high in 3 clusters. The CAST score and the number of joints per month were strongly correlated. However, it should be noted that one-third of the lowest users had high CAST scores. It is more difficult to identify an at-risk user if (s)he consumes little in terms of quantity and frequency. This difficulty particularly applies because only 2% of GPs used questionnaires to assess risky cannabis use [12]. This study provides encouragement to GPs to use the CAST score, which can be used to screen risky users regardless of the amount of cannabis use.

### Strengths and limitations of the study

The sample itself is both a strength and weakness of this study. Participants were selected by their GPs for the research study, and the sample may therefore not be representative of all user patients. The distribution revealed a majority of daily users (46.2%, n = 121), whereas in France in 2017, daily users represented 4% of adolescent 17 year-olds and 8% of young adults (18–25)[3]. This difference can be explained by the effect of the study. Participating in a research study can modify the behavior of both the patient and the GP [34][35]. On the one hand, GPs could have included patients whose needs were more severe for the subject studied, as was the case in the CANABIC study [14] and other studies with the same design [36]. However, ours was a pragmatic study in that the GPs proposed the patients to whom they would have proposed screening and intervention in real life. On the others hand, we might fear a response bias: patients could have underreport information about their cannabis use, but the concordance between the answers to the GPs and the anonymous answers was good, and suggests that they answered objectively to the GP. The age of onset of use, 15.15±1.9 years, was consistent with the national age of cannabis initiation (15.3 years) [4]. The male-to-female ratio in our study was 2:3. Boys are more likely to use cannabis than girls in Europe as a whole [2]. This difference is particularly true in France, where the proportion of regular users is double for boys compared with girls (4.5% vs 9.7%) between 17 to 25 years old [4][10]. However, there was no difference in the distribution of boys and girls in the use groups or clusters.

This study shows that by interviewing all patients in routine care for recruitment to a research study, GPs identified cannabis users with very different profiles, different levels of severity of use, and who responded honestly about their cannabis use. The different profiles that had been identified should encourage GPs to ask systematically their patients from 15 to 25 years of age about their use of cannabis. Then, the GPs could identify risky profile (e.g., that could help him/her to tailor the package of care specifically for each group of patients.

## Supporting information

S1 Table STROBE Statement. (PDF)

S2 Table Trends for the main criteria according to each cluster. (PDF)

S1 Appendix GP questionnary. (PDF)

S2 Appendix Self questionnary. (PDF)

## Abbreviations

BI: Brief intervention
CANABIC: CANnabis And young users: A Brief Intervention to reduce their Consumption
CAST: Cannabis abuse screening trial
ENT: Ear Nose Throat
FRAMES: Feedback, responsibility, advice, menu, empathy, self-efficacy
GP: General practitioner
MCA: Multiple correspondence analysis
OFDT: French Observatory of Drugs and Addictions
