# Supplementary material for: Cannabis users: Screen systematically, treat individually. A descriptive study of participants in a randomized trial in primary care
Source: PLoS One. 2019 Dec 2;14(12):e0224867. doi: 10.1371/journal.pone.0224867 (PMC6886842; doi:10.1371/journal.pone.0224867)
Supplement: S2 Table — (PDF) [file pone.0224867.s002.pdf]

Supplemental information. Trends for the main criteria according to each cluster.

	Risky Adolescent users	Risky young unemployed users	Risky young worker users	Low risk student users	Low risk Adolescent users
	C1 (38)	C2 (37)	C3 (41)	C4 (25)	C5 (58)
Joints / month, mean(sd)	33.5 [15-90]	45 [25-100]	30 [20-60]	5 [3-10]	6 [3-20]
CAST	≥3	≥3	≥3	<3	<3
Age, years	<21	>21	>21	ns	<21
Age of onset use, years	<16	ns	<16	ns	ns
Alone use	yes	yes	yes	no	no
Use on week day	yes	yes	yes	no	no
Profesionnal status	na	Unemployed	Workers	Students	na
Motivation to use	to fall asleep by habits	to fall asleep against anxiety by habits	against anxiety by habits	to party	ns
Psychotropic treatment	yes	yes	no	no	no
Drive	yes	yes	yes	no	no
Cocaine use	no	no	yes	no	no

na : not adapted; ns : not significant
