# Supplementary material for: Cannabis users: Screen systematically, treat individually. A descriptive study of participants in a randomized trial in primary care
Source: PLoS One. 2019 Dec 2;14(12):e0224867. doi: 10.1371/journal.pone.0224867 (PMC6886842; doi:10.1371/journal.pone.0224867)
Supplement: S1 Appendix — (PDF) [file pone.0224867.s003.pdf]

1st Consultation : M0

Date of consultation : |__|__| |__|__| |__|__|

Reason of consultation : ....

Sociodemographic information

A.1.Occupational status

Active ☐

Job seeker ☐

Disability ☐

Without profession ☐

Student ☐

Other ☐

Specify : ....

A.2.Socioprofessional category

Farm operator ☐

Craftsman, shopkeeper ☐

Business manager

Executive, liberal profession☐

Employee ☐

Worker ☐

Other ☐

Specify:

A.3.Family situation

Married – in a marital way ☐

Single ☐

Widowed ☐

Divorced, separated ☐

Other ☐

Specify :

A.4.Housing

Lives alone	☐ yes	☐ no
Share home with others (roommate, family)	☐ yes	☐ no

B.1.History

Surgical history : ☐ yes ☐ no, if yes, specify : ....

....

Medical history : ☐ yes ☐ no, if yes, specify : ....

....

Family history : ☐ yes ☐ no, if yes, specify : ....

....

B.2. Ongoing psychotropic treatment :

Drug name (commercial name, specify if generic drug)	Daily dosage	Start date (DD/MM/YYYY)
....	....	....
....	....	....

Information on the patient’s cannabis use - M0

1 – In the month preceding this consultation, the patient has used cannabis as :

1.1. Joint : ☐ yes ▶ Number of joint(s) per month |__|__|__|

☐ no

1.2. Bong or water pipe : ☐ yes ▶ Number of bong(s) per month |__|__|__|

☐ no

2 – The patient has used cannabis as : ☐ Resin

☐ Herb

☐ Oil

☐ Synthetic cannabis

3 - Age at first use of cannabis : |__|__|

Information on the use of other substances

4 – In the month preceding, the patient has been drinking alcohol :

☐ yes ▶ Number of glass(es)/week : |__|__|__|

☐ no

5 - In the month preceding, the patient has been smoking tobacco:

☐ yes ▶ Number of cigarette(s)/week : |__|__|__|

☐ no

6 – Other toxic substances :

<u>During his life</u>

7 – Has the patient ever experienced heroin use ? : ☐ yes ☐ no

8 - Has the patient ever experienced cocaine use ? : ☐ yes ☐ no

9 - Has the patient ever experienced use of other illicit drugs ? ☐ yes ☐ no

9.1. If yes, which ones : ....

Achievement of the Brief Intervention- M0

10 – Duration of the consultation : ....
