# Supplementary material for: Cannabis users: Screen systematically, treat individually. A descriptive study of participants in a randomized trial in primary care
Source: PLoS One. 2019 Dec 2;14(12):e0224867. doi: 10.1371/journal.pone.0224867 (PMC6886842; doi:10.1371/journal.pone.0224867)
Supplement: S2 Appendix — (PDF) [file pone.0224867.s004.pdf]

CANABIC Study

Please complete this questionnaire, place it in the « T » envelope,
seal the envelope and give it to your general practitioner

Date |__|__| |__|__| |__|__|

In the past month

- How many joints have you smoked ? : ....
- How many bongs did you consumed ? : ....

How do you consume : *(Several answers are possible)*

Who do you smoke with ?

- alone ☐
- with friends ☐

When do you smoke ?

- the week ☐
- the week-end ☐

- during the day ☐
- in the evening ☐

Where do you smoke ?

- at home ☐
- at friends' house ☐
- in your workplace ☐
- in a nightclub ☐
- other ☐

Why do you smoke ?

- to relax ☐
- to party ☐
- to reduce anxiety ☐
- to sleep ☐
- by habit ☐

How do you get your cannabis ?

Own cultivation ☐
Purchase ☐
I don't have my own cannabis : shared use ☐

What is the approximate cost of your own consumption ? ________ € /month

Your opinion

Do you think that smoking cannabis :

- could cause health problems for you ? ☐ Yes ☐ No
- may have an impact on your personal life ? ☐ Yes ☐ No
- may have an impact on your professional life ? ☐ Yes ☐ No

Driving

Would you drive after smoking cannabis ? ☐ Yes ☐ No

Please turn the page! →

About~~round~~ your consumption

Have you ever smoked cannabis before noon ? ☐ Yes ☐ No

Have you ever smoked cannabis when you were alone ? ☐ Yes ☐ No

Have you had any memory problems when you smoke cannabis ? ☐ Yes ☐ No

Have any friends or family members ever told you that you should reduce your cannabis use? ☐ Yes ☐ No

Have you ever tried to reduce or stop your cannabis use without success? ☐ Yes ☐ No

Have you ever had any problems because of your cannabis use (quarrel, fight, accident, bad school results...)? ☐ Yes ☐ No

Thank you for answering these questions.

CANabic
